# Diffusion in Molten Sodium Carbonate

**DOI:** 10.1021/acs.jpca.4c04649

**Published:** 2025-02-08

**Authors:** M. C. Wilding, F. Demmel, M. Wilson

**Affiliations:** † UK Catalysis Hub, Research Complex at Harwell, Rutherford Appleton Laboratory, Didcot OX11 0DE, U.K.; ‡ ISIS Facility, Rutherford Appleton Laboratory, Didcot OX11 0QX, U.K.; § Physical and Theoretical Chemistry Laboratory, Department of Chemistry, University of Oxford, South Parks Road, Oxford OX1 3QZ, U.K.

## Abstract

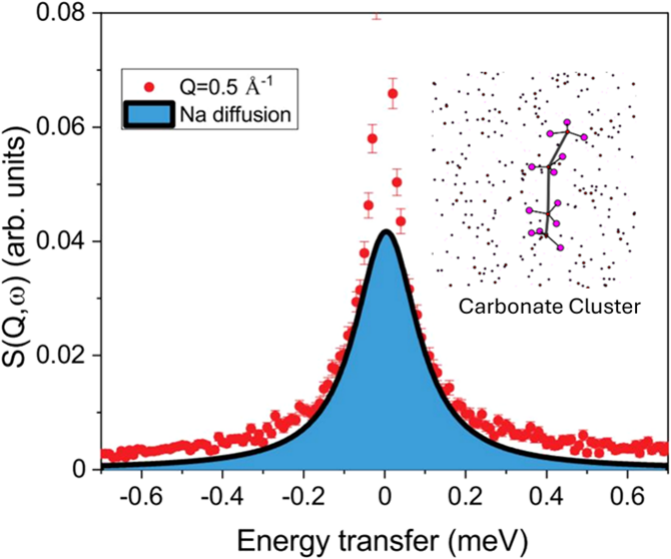

The diffusion of sodium and carbonate ions in molten sodium carbonate is investigated by quasi-elastic neutron scattering (QENS) at *T* = 1143 K. The quasi-elastic scattering at small wave vectors is dominated by diffusing sodium ions, and the derived self-diffusion coefficient of *D*_Na_ = 4.5 × 10^–5^ cm^2^/s agrees well with previous tracer diffusion measurements. The quasi-elastic scattering from the carbonate anion is coherent, and the coherent scattering dominates the QENS signal at scattering vectors with a modulus greater than 1 Å^–1^. The line width of the coherent scattering function is used to obtain the diffusion coefficient of the carbonate anion at this temperature of *D*_CO_3_^2–^_ = 2.4 × 10^–5^ cm^2^/s, again in agreement with values from tracer diffusion studies. The results from this QENS measurement are larger compared with molecular dynamics simulations using a recently developed model, which introduces flexibility to the carbonate anion and allows charge to fluctuate across the anion. The model was improved concerning the melting point of the simulated liquid. Scaling the temperature in terms of this melting point is shown to bring the simulated and experimental diffusion coefficients into good agreement. The self-diffusion coefficients are consistent with those expected for a fragile liquid, and the changes in viscosity expected as the carbonate liquid is cooled are explained by the development of chains and complex structures that directly result from the flexibility of the anion introduced in this modeling approach. This simulation methodology can therefore be applied to further studies of complex molten salts.

## I Introduction

Molten salts are seeing a renaissance from an application point of view. The large heat capacities and large liquid temperature range make molten salts attractive as heat storage and transport media, e.g., for heat storage in modern solar power plants or in next-generation nuclear reactors.^1,2^ There are many potential applications of molten carbonates, for example, in fuel cells and in CO_2_ sequestration. In addition, many important rare-earth elements are highly soluble in molten carbonates (e.g., ref (3)), so an understanding of the structure and dynamics of the pure liquid is key to future studies in this area. Accordingly, there has been revived interest in carbonates, taking advantage of the advances in experimental and computational techniques that have emerged in recent decades. An understanding of the structure and dynamics of molten salts on an atomic and molecular level is critical if they are to have strategic applications.

The study of molten salts has a long tradition in theory, experiment as well as simulation, see for example, refs (4−6). On the computational side, a lot of effort was devoted to elucidate the microscopic dynamical foundations of transport parameters. First simulations on molten salts used classical rigid ion potentials to derive transport coefficients.^7^ In parallel, the inclusion of polarization effects was achieved, which resulted in an increase of the diffusion coefficient for the cation.^8^ Later on, several studies assessed the influence of polarization on the ion dynamics^9,10^ and also *ab initio* methods have been applied, see, for example, refs (11−13). The development of advanced simulation models, in which the molecular anion charge is allowed to vary according to the environment, indicates that, far from being simple ionic liquids, molten carbonates show a rich structure with significant temperature-dependent intermediate-range ordering.^14^

Even in a chemically simple liquid carbonate such as Na_2_CO_3_, the total scattering pattern is formed from the weighted sum of six partial pair contributions, and interpretation of the structure requires a detailed atomistic model. For carbonates, the simplest models assume the CO_3_^2–^ anion as retaining a fixed geometry with fixed charge distribution, which sum to give the required formal −2 charge. More advanced models allow both flexible molecular geometries and for the charges on each site to fluctuate according to the environments. The charge separation between the atoms in the molecular anion is driven by their respective electronegativities, and for simple anions such as carbonates, a single metric, the difference in the charge held by the O and Δ atoms (Δ*q*) defines the charge separation. These more flexible models have helped uncover previously hidden rich structural behavior.^15,16^ The charge separation is found to influence the separation of the distinct peaks in the total scattering pattern and leads to the emergence of a secondary length scale and an increase in fragility as Δ*q* is increased. At high Δ*q*, the high-temperature liquids are characterized by isolated carbonate anions, but as the temperature decreases, more extensive low-dimensional chain-like carbonate structures are formed, concomitant with the emergence of a secondary length scale. In studies of the carbonate K–Mg glass,^17^ the structure and dynamics of the glass-forming liquid is determined by the strong Coulomb interaction between potassium and the distorted carbonate anion. The flexibility of the carbonate anion is demonstrated unequivocally through ^13^C MAS NMR measurements made on K–Mg glass.

Diffusion in solids, liquids, and gases is driven by concentration gradients, according to Fick’s law, and macroscopic measurements use this principle to determine diffusion coefficients by using tracer techniques. At long length scales, in the hydrodynamic regime, self-diffusion can be represented by a partial differential equation for a tagged particle with the solution of a Lorentzian line shape in energy space. The half-width at half-maximum (HWHM) of the Lorentzian peak is related to the diffusion coefficient *D* by HWHM = Γ = ℏ*DQ*^2^, where *Q* is the wave vector. This relationship is probed directly by quasi-elastic neutron scattering (QENS). QENS has become a powerful method in the study of single particle dynamics, especially for liquids where disturbance from convection can be avoided. For example, the incoherent scattering cross section of sodium has been used in self-diffusion studies of molten alkali halides.^10,18−20^ The results of QENS measurements can be directly compared with the results from molecular dynamics simulations, and hence, a QENS study of Na_2_CO_3_ is an ideal benchmark of the flexible anion fluctuating charge model for carbonates. There are few experimental studies of the microscopic dynamics of sodium carbonates due to the relatively high melting temperatures and reactive nature of the carbonates. In this contribution, we will experimentally determine the diffusion coefficients of the sodium and carbonate components of Na_2_CO_3_ and compare these data with existing diffusion measurements and the results of simulations that utilize both fluctuating charges and flexible anions.

## II Experimental and Computational Details

The QENS experiment was performed at the ISIS Facility U.K., using the OSIRIS spectrometer.^21^ Na_2_CO_3_ powder was dried for 24 h and then was filled into an annular niobium can with a 3 mm gap with a wall thickness of 0.4 mm. The can was then sealed by electron beam welding. Niobium is a nearly perfect coherent scatterer and will not contribute to the elastic line except where Bragg refections appear. The first reflection of niobium is at *Q* = 2.7 Å^–1^, which is outside the wave vector range used during this experiment; therefore, the empty can contribution in this setup will be very small. The sealed cell was installed into a standard furnace with niobium shields. QENS data for molten Na_2_CO_3_ were collected at a temperature of *T* = 1143 K, above the melting point (*T*_melt_ = 1127 K^22^). The temperature uncertainty during the measurements was smaller than ±1.5 K. Empty cell runs were performed at a temperature of 1073 K.

**Figure 1:**
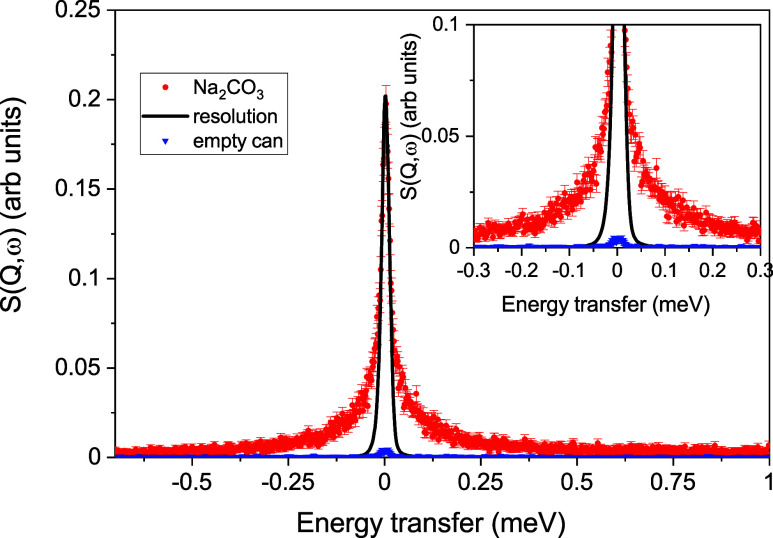
Spectrum shown at *Q* = 0.5 Å^–1^ together with the empty cell scan and the peak normalized resolution function. The inset shows an enlarged view of the quasi-elastic region.

The QENS experiment at OSIRIS used an end energy of *E*_f_ = 1.845 meV with the energy resolution, determined from a vanadium measurement, of fwhm = 0.025 meV. The covered wave vector range is 0.25 Å^–1^ < *Q* < 1.8 Å^–1^ and energy transfers between −0.7 and 1.5 meV were recorded. About 8 h of beam time was used for the sample and a similar time for the empty cell measurement.^23^ In Na_2_CO_3_, only sodium has a sizable incoherent neutron cross section.^24^ The incoherent cross section of a Na_2_CO_3_ molecule is σ_inc_ = 3.24 barn and the coherent cross section is σ_coh_ = 21.56 barn, hence much larger than the incoherent one. However, the quasi-elastic scattering at the small wave vector stems mainly from the diffusing sodium ions due to the small liquid structure factor at small wave vectors. The measured total intensity is a product of the scattering cross sections with the structure factor. The structure factor for coherent scattering has very small values toward zero wave vector and increases dramatically when the first structure factor maximum is approached. The carbonate ions will scatter only coherently and the intensity will follow the structure factor of molten Na_2_CO_3_, with a first structure peak around *Q* ≈ 1.6 Å^–1^.^16^ Below *Q* ≈ 1 Å^–1^, the coherent contribution to the scattered signal can be regarded as small. Nevertheless, the coherent part is not completely negligible even in the small wave vector range. Hence, any coherent scattering makes only a small contribution and is neglected in our data analysis, and therefore, we can fit the spectra with a single Lorentzian to describe the diffusive motion of the sodium ions in a *Q*-range up to 1 Å^–1^. When the structure factor maximum is approached, the coherent cross section of the carbonate (the carbonate has no incoherent scattering) in combination with the structure factor maximum dominates the scattering. The incoherent scattering cross section from the sodium ions is about 15% of the coherent cross section and multiplied with the structure factor can be neglected in this Q-range. A similar argument applies to the coherent scattering from the sodium ions. To summarize, due to the interplay of cross sections and structure factor, we are able to extract diffusion coefficients for sodium and carbonate in separate regions of wave vectors. Figure 1 shows a spectrum of molten Na_2_CO_3_ at *T* = 1143 K for *Q* = 0.5 Å^–1^. The empty niobium cell clearly contributes only a small amount to the signal at this wave vector. Included is the energy resolution from a vanadium measurement, which is much smaller than that of the measured spectra from the molten salt. The inset zooms into the quasi-elastic region, which demonstrates a strong quasi-elastic signal from the diffusing sodium ions. In addition to the quasi-elastic signal, there is an elastic contribution.

The data analysis included monitor normalization, a detector efficiency correction, and empty cell subtraction. A constant energy binning of 0.002 meV has been applied. With the chosen sample dimensions, the transmission will be about 0.88 and an estimate for a rod with a similar scattering geometry gives a 9% contribution for twice scattered neutrons.^25^ No attempt was made to correct for this small multiple scattering contribution. For modeling the obtained spectra, a sum of a δ-function for the elastic part and a single Lorentzian for the quasi-elastic intensity was used. This model function was convoluted with the measured resolution function and a linear sloping background was added before fitted to the data. The Mantid framework was used for all data analysis steps.^26^

In addition, MD simulations have been performed to describe the diffusion properties of molten Na_2_CO_3_ over a wide temperature range. A recently developed flexible anion model in which the charges on the C and O atoms are allowed to vary was applied.^16^ In order to assess the effect of the molecular anion, charge distribution simulations are performed on a sodium carbonate liquid with different charge distributions. The ion diffusion coefficients are calculated for each potential model from the respective mean-squared displacements. More details about the simulations can be found in ref (16) and a summary is presented in the Supporting Information (section II). Previous simulations demonstrated a slower diffusion than that measured with tracer diffusion experiments. Here, we improve these simulation results with a more profound study.

## III Results and Discussion

**Figure 2:**
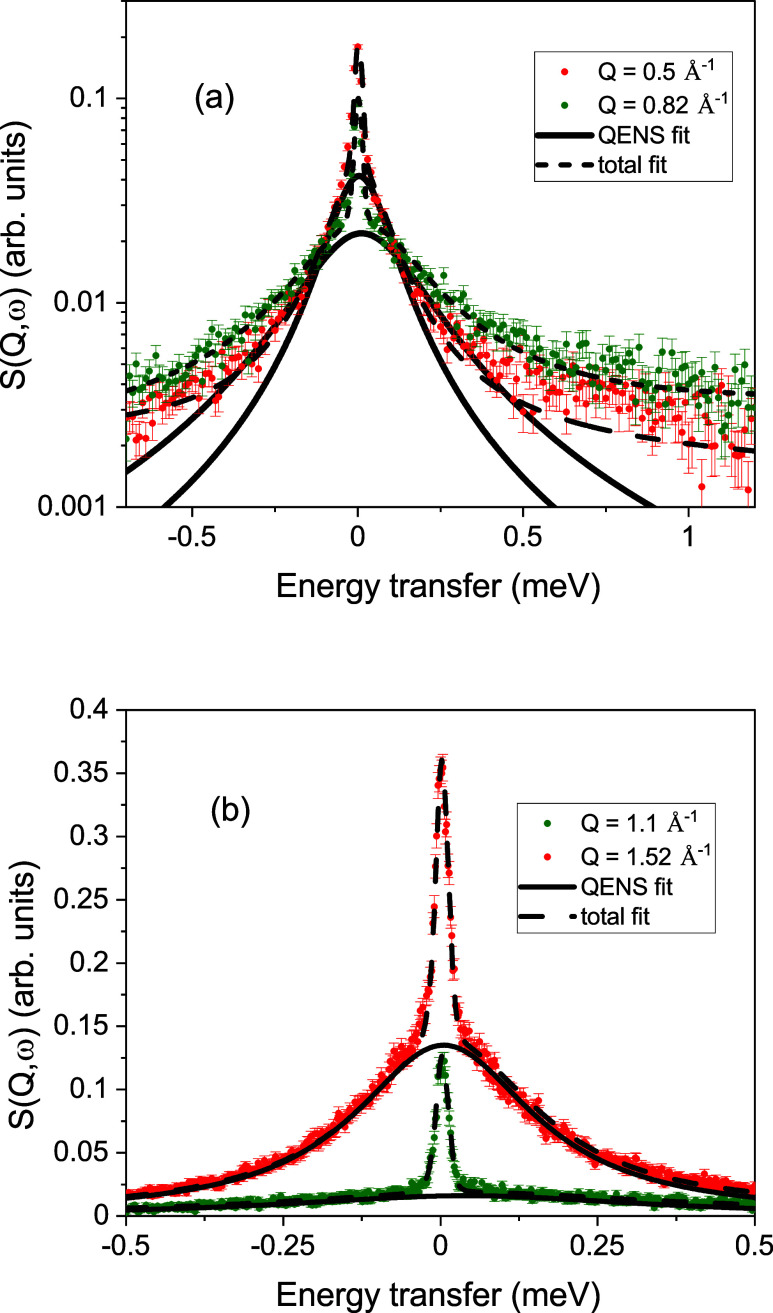
(a) Two spectra for different wave vectors plotted on a logarithmic scale to demonstrate the spectra evolution with increasing *Q*. (b) Spectra at larger *Q* vectors toward the structure factor maximum on a linear scale. Included are the fits to the quasi-elastic signal as a solid line and the total fit as a dashed line.

**Figure 3:**
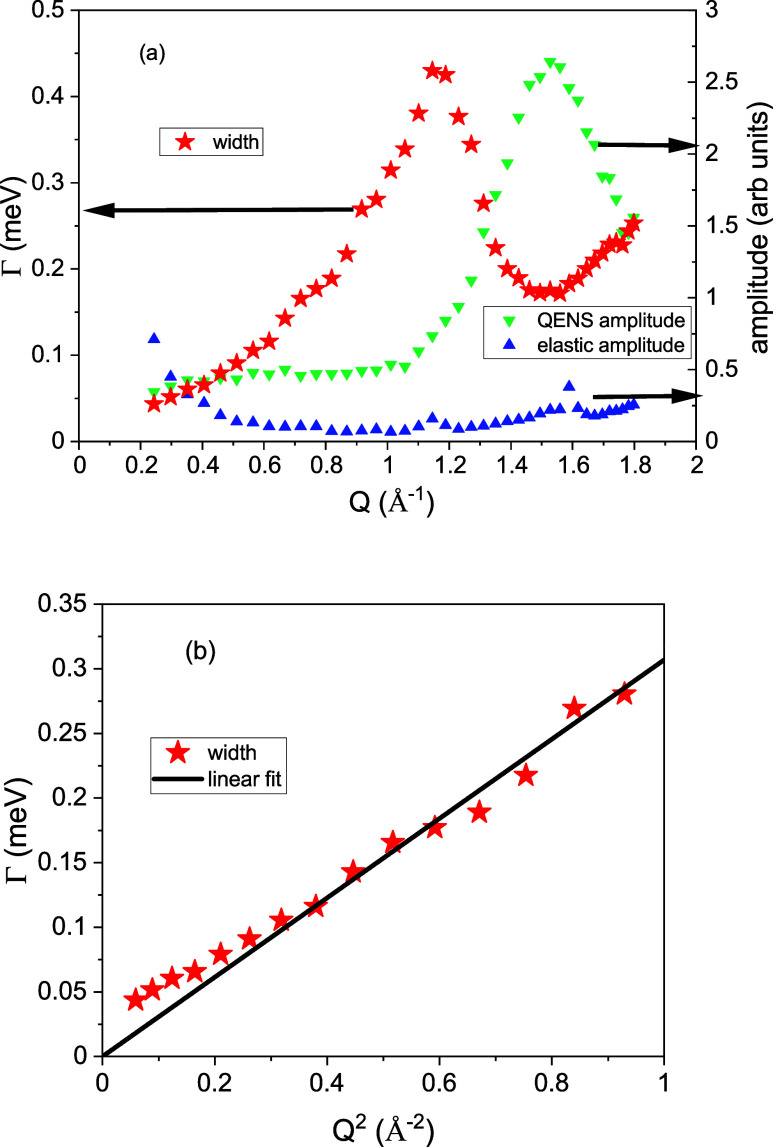
(a) Widths from the experimental data plotted against *Q*. Included are the amplitude values for the QENS signal and amplitudes of the elastic contribution. (b) Widths from spectra up to *Q* < 1.0 Å^–1^ plotted against *Q*^2^. A fit with a line is included to obtain the diffusion coefficient.

The results of the single temperature Na_2_CO_3_ QENS measurement are shown for two *Q* values in Figure 2a. The spectra at *Q* = 0.5 Å^–1^ and *Q* = 0.82 Å^–1^ are plotted on a logarithmic intensity scale, and the full lines show the quasi-elastic contribution to the fit. Also shown are the total fits as a dashed line. With increasing wave vector, the width is increasing as expected for a translational diffusion process. The *Q*-dependence of the QENS spectra for the entire range of *Q* values is shown in the Supporting Information (Figure S1). In panel (b), the spectra of two larger wave vectors *Q* = 1.1 Å^–1^ and *Q* = 1.52 Å^–1^ are plotted on a linear scale. The spectrum at *Q* = 1.52 Å^–1^ approaches the structure factor peak of the molten salt. The first peak in the structure factor for molten Na_2_CO_3_ occurs at *Q* ≈ 1.6 Å^–1^. There is an increase in the quasi-elastic intensity, which evidences the increased contribution of the coherent scattering of the moving carbonate ions to the QENS signal. From the figure, it is already apparent that the width at *Q* = 1.52 Å^–1^ is smaller than the spectrum at *Q* = 1.1 Å^–1^, the signature of the deGennes narrowing. The relationship between the extracted widths HWHM = Γ(*Q*) of the Lorentzian fit and wave vector *Q* is shown in Figure 3. In panel (a), the width for the whole measured wave vector range is plotted. The widths show a distinct minimum around the structure factor maximum *Q* ≈ 1.6 Å^–1^. This reduction in the line width of a liquid is known as deGennes narrowing.^27^ The amplitude of the quasi-elastic signal is included in the figure, and it follows the structure factor. This correlation shows that the quasi-elastic signal at a larger *Q* is dominated by the collective dynamics. The maximum intensity is correlated with the minimum width.

The widths of the Lorentzian peaks show a parabolic increase with *Q*, characteristic of self-diffusion, at values smaller than the structure factor maximum. Hydrodynamics predicts for the diffusion of a tagged particle a line width broadening proportional to *Q*^2^ with a proportionality coefficient D:^6^ Γ = ℏ*DQ*^2^. This relation is only applicable to the self-particle dynamics, and therefore, the coherent contribution should be kept negligibly small through restricting the evaluation of the sodium diffusion coefficient to *Q*-values smaller than 1.0 Å^–1^. Then, the diffusion coefficient can be obtained by plotting the widths of the quasi-elastic peaks against *Q*^2^, and a linear fit yields a value of 4.5 ± 0.28 × 10^–5^ cm^2^/s (see Figure 3b). The widths deviate from the line toward larger values at small *Q* vectors, which might be due to the not corrected multiple scattering or the influence of the small coherent contribution.

Figure 3a also includes the amplitude of the fitted elastic intensity. A possible reason for this contribution might be a corrosive interaction between the sodium carbonate liquid and the niobium container. The main commercial sources of niobium are associated with so-called carbonatite deposits (e.g., Elliott et al.^3^). Although niobium ore is not a carbonate, niobium and other rare-earth elements are soluble in carbonates. A more intriguing possibility for this elastic intensity is that a slow structural relaxation process occurs, which is too slow to be resolved by using this spectrometer. Simulation work is underway to investigate this possibility. Note that this additional elastic intensity has no influence on the obtained results.

At wave vectors larger than *Q* ≈ 1 Å^–1^, coherent scattering starts to dominate the measured signal (see Figures 2(b) and 3(a)). Coherent neutron scattering provides insight into the collective movements of the particles. The quasi-elastic line shows a narrowing when the structure factor reaches its maximum, the deGennes narrowing.^27^ This behavior can be understood by the fact that a density fluctuation needs more time to relax on the next-neighbor length scale because this process involves a rearrangement of the surrounding particles in a dense liquid. This longer relaxation time corresponds to a reduction in the frequency of the line width. Within a kinetic theory for a dense fluid, the line width of the scattering function at the structure factor maximum has been related to a self-diffusion process of a caged particle, which enables the density fluctuations to decay.^28^ This formulation provides a connection between the Enskog self-diffusion coefficient *D*_E_ of a hard-sphere fluid and the measured half-width at half-maximum (HWHM)^28^
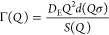
1where *d*(*Q σ*) = (1 – *j*_0_(*Q*σ) + 2*j*_2_(*Q*σ))^−1^ is given by a combination of spherical Bessel functions *j* of order 0 and 2 and σ denotes the hard-sphere diameter of the moving particle. This relation strongly resembles the hydrodynamic description of the self-diffusion process. However, the structure factor *S*(*Q*) takes into account the slowing of the diffusion process at next-neighbor distances. This methodology has successfully been applied to liquid aluminum and molten NaBr.^29,30^

This formalism is applied to the coherent quasi-elastic signal to evaluate the movements of the carbonate ions, neglecting the small incoherent contribution at the structure factor maximum and the coherent scattering from the sodium ions. From the fit of the widths, we obtain a minimal width Γ = 0.17 ± 0.01 meV (see Figure 3); for the structure factor maximum, we use the maximum value from the carbon–carbon partial structure factors from the simulation *S*_CC_(*Q* = 1.6 Å^–1^) ≈ 3.0,^16^ and for the hard-sphere diameter σ of the carbonate ions, we use σ = 4 Å. The variation of *S*_CC_ with charge separation is shown in the Supporting Information (Figure S2). This value was calculated from the CO_3_^2–^ ion volume^22^ and agrees well with partial pair correlation function simulations.^16^ We obtain a carbonate diffusion coefficient of *D*_CO_3_^2–^_ = 2.4 ± 0.15 × 10^–5^ cm^2^/s. This evaluation is based solely on binary collisions of the particles and neglects all memory effects within the diffusion process.

**Figure 4:**
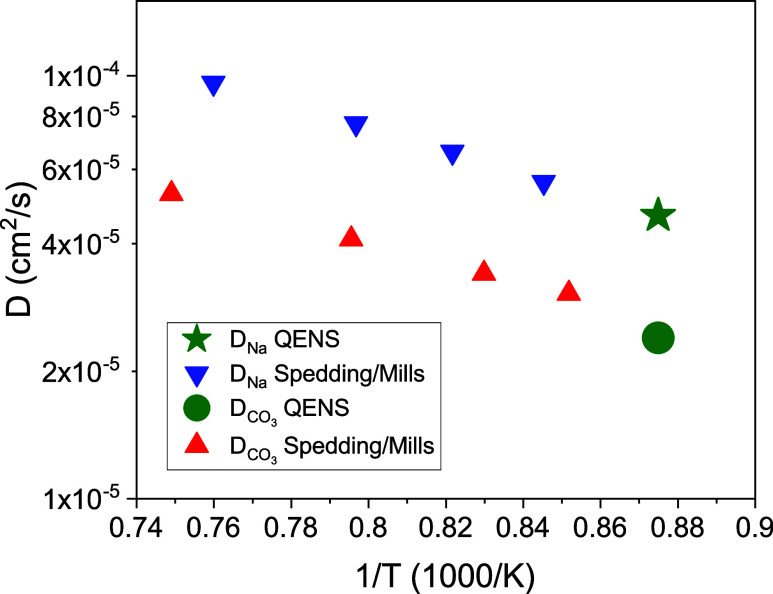
Diffusion coefficients for sodium and carbonate ions are plotted in an Arrhenius-type plot. The error bars are smaller than the symbol size. Literature values from tracer ion diffusion experiments are included.^31^

In Figure 4, we plot the diffusion coefficients from our measurements for sodium and carbonate ions in an Arrhenius-type plot against the inverse temperature. Included are literature data from tracer diffusion measurements (triangles).^31^ Our deduced diffusion coefficients for the sodium ions agree very well with the literature data, if one assumes an activated process for the diffusion steps with a single activation energy. The carbonate diffusion coefficient seems to coincide with the expected temperature behavior from the tracer diffusion measurements despite its approximative derivation. Due to the approximative nature of this evaluation, we consider the good agreement as fortuitous. We conclude that the results from the QENS experiment show good agreement with the previous macroscopic tracer diffusion measurements.

**Figure 5:**
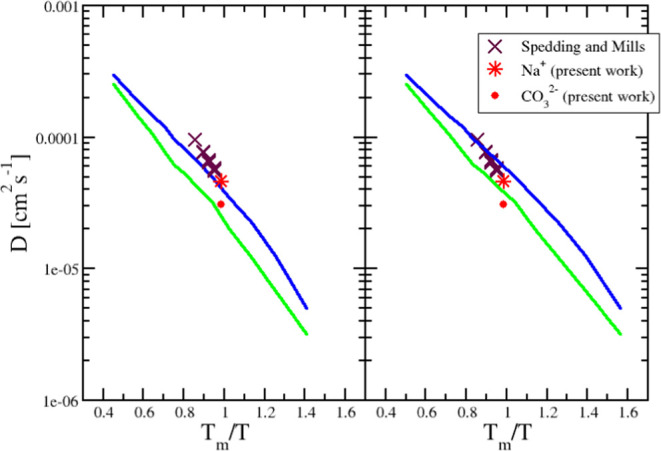
Diffusion coefficients for sodium ions from the MD simulation shown in an Arrhenius-type plot against a normalized inverse temperature (circle + line). Literature values from tracer ion diffusion experiments^31^ (triangle) and *D*_Na_ from the QENS experiment (star) are included.

Direct initial comparison with the MD simulation results shows significant differences. The QENS-measured values of *D*_Na^+^_ = 4.6 × 10^–5^ cm^2^s^–1^ and *D*_CO_3_^2–^_ = 2.4 × 10^–5^ cm^2^s^–1^ are larger than the respective values from the MD simulation.^16^ A possible reason for this difference might be that the simulation model does not reproduce the experimental melting point (*T*_m_ ∼ 1143 K). Reproducing temperature-driven thermodynamic transitions is notoriously difficult, as the respective solid- and liquid-state chemical potentials will be near-parallel around the melting point. As a result, relatively small changes in the underlying potential energy functions can lead to large shifts in the observed melting points. The melting point predicted by the model is determined by generating a (tetragonal) simulation cell that contains both the crystalline and liquid phases separated by two boundaries in order to facilitate the use of periodic boundary conditions. The simulation cell temperature is then increased systematically from *T* = 1000 K, in steps of Δ*T* = 25 K, with simulation time scales of the order of *t* ∼ 1 ns at each temperature. The melting of the crystalline region of the simulation cell is characterized by a near-first-order change in the potential energy profile, from which the melting temperature is estimated as *T*_mp_^sim^ ∼ 1250 ± 25 K. Figure 5 shows the effect on the ion diffusion coefficients of scaling the temperature axis with the ratio of this melting temperature to that observed experimentally. The left panel shows the raw diffusion data obtained at the two limiting molecular anion charge distributions studied, thus giving the range of values obtainable with the present model, while the right panel shows the effect of the temperature scaling. The scaling brings the simulated diffusion coefficients into a far better agreement with the experimental data. Depending on the charge distribution model, the experimental diffusion coefficients can be reproduced, and from these data a more accurate conclusion about the fluctuating charge potential can be derived. There are differences in the slopes of the simulated and the experimental diffusion coefficients, which could indicate a deficiency in the potential model, resulting in a difference in activation energy.

A recent molecular dynamics simulation of Li–K carbonate mixtures^34^ was performed by introducing polarization effects. The calculated self-diffusion coefficients for lithium and potassium at 923 K are similar to that of sodium at a similar temperature.^16^ The simulated carbonate diffusion coefficient is a factor of 3 smaller than the alkali ion *D* values, which compares reasonably well with a factor of 2 in our measurement.

From Figure 5, it is evident that the diffusion coefficients demonstrate a non-Arrhenius-type behavior in the supercooled state, which is emphasized by the charge difference in the model. Through the Stokes–Einstein relation, the diffusion coefficient is directly related to the viscosity. Therefore, the simulated *D* values might indicate non-Arrhenius behavior of the viscosity. Carbonate liquids are fragile^35^ as demonstrated by differential scanning calorimetry (DSC) measurements made on the 0.55K_2_CO_3_-0.45MgCO_3_ glass discussed above. Fragile liquids show a non-Arrhenius viscosity-temperature relation.^35^ This means that the activation energy for viscous flow will be greater close to the calorimetric glass transition than in the stable, high-temperature regime, which is more amenable to conventional viscosity measurement. The viscosity of Na_2_CO_3_ has been evaluated in a study^36^ based on the values of *D*_Na^+^_ and *D*_CO_3_^2–^_ obtained from the molecular dynamics simulations of Wilson et al.^16^ combined with measurements of viscosity for mixed alkali carbonates.^32,33^ It is concluded that the fragility of Na_2_CO_3_ is close to that of (40CaNO_3_-60KNO_3_), which is generally considered to be the archetypical fragile liquid. The simulations indicate the formation of chains and other complex structures as the liquids are supercooled and the associated increase in viscosity is a direct reflection of the flexibility of the carbonate anion and fluctuating charge distribution.

## IV Conclusions

A quasi-elastic neutron scattering experiment performed on molten Na_2_CO_3_ at *T* = 1143 K has been used to determine the self-diffusion coefficients of sodium and carbonate ions in this increasingly important molten salt. The quasi-elastic scattering at small wave vectors yields the self-diffusion of the sodium ions and the value is in good agreement with that determined by tracer diffusion. At larger wave vectors, around the structure factor maximum, the quasi-elastic signal is dominated by the motions of the carbonate ions. This quasi-elastic scattering can be interpreted as a diffusion motion, which allows the diffusion coefficient for the carbonate ions to be extracted, too. The diffusion coefficient for the carbonate ion is also in good agreement with previously performed tracer diffusion measurements. The experimentally derived values of sodium and carbonate diffusion are compared with values obtained from molecular dynamics simulation. An improved MD simulation model that allows the flexibility of the carbonate anion and fluctuation of charge across the anion provides diffusion coefficients that are in good agreement compared with these experimental results. Although the QENS data only provide detail in the high-temperature regime, the simulations indicate that the carbonate liquids have strong temperature-dependent dynamics consistent with the carbonates and other molten salts being fragile liquids. This satisfactory agreement between simulation and experiment opens up new opportunities for studying the structure and dynamics of complex molten salts.

## Supporting Information Available

Further 10 quasi-elastic neutron scattering spectra illustrating the changes in QENS spectra with *Q*-vector; quasi-elastic contribution to these spectra shown as a full line; simulation details for the fluctuating charge model (FCM) that has been used in the molecular dynamics simulation of the sodium carbonate liquids; and influence of different charge distributions (Δ*q*) used in the FCM on the carbon–carbon partial structure factor (*S*_CC_(*Q*)) (PDF)
