# Supplementary material for: Diffusion in Molten Sodium Carbonate
Source: J Phys Chem A. 2025 Feb 8;129(7):1890–5. doi: 10.1021/acs.jpca.4c04649 (PMC11848922; doi:10.1021/acs.jpca.4c04649)
Supplement: Supplementary file 1 — jp4c04649_si_001.pdf [file jp4c04649_si_001.pdf]

Diffusion in molten sodium carbonate. Supplementary Information.

M. C. Wilding,[1] F. Demmel,[2] and M. Wilson[3]
[1)] *UK Catalysis Hub, Research Complex at Harwell, Rutherford Appleton Laboratory, Didcot, OX11 0DE, UK*
[2)] *ISIS Facility, Rutherford Appleton Laboratory, Didcot, OX11 0QX, UK*
[3)] *Physical and Theoretical Chemistry Laboratory, Department of Chemistry, University of Oxford, South Parks Road, Oxford OX1 3QZ, UK*

I. EXPERIMENTAL SPECTRA

Figure S1 shows ten further experimental spectra over the whole explored Q-range. Included are the total fit (dashed line) and the quasielastic contribution (full line), both convoluted with the resolution function. The inset displays the whole spectrum whereas the main figure focuses on the quasielastic signal only. Please note that the intensity axis varies according to the scattered intensity with the Q-vector. The origin for the quasielastic intensity changes with the Q-vector from the sodium diffusion at small Q to the coherent quasielastic scattering of the carbonates at larger momentum transfers.

II. SIMULATION DETAILS.

A fluctuating charge model (FCM) is employed which incorporates both a flexible description of the CO_3^{2-} molecular anion and the internal charge distribution (see ref.[1] and references therein). In a FCM the electronegativity, $\chi_{i\alpha}$, on an atomic site α within a given molecule i is given by

$$\chi_{i\alpha} = \chi^0_{i\alpha} + J^0_{\alpha\alpha} q_{i\alpha} + \sum_{\beta\neq\alpha} J_{\alpha\beta} q_{i\beta} + \sum_{j\neq i}\sum_{\beta} \frac{q_{j\beta}}{r}, \qquad (1)$$

where $q_{i\alpha}$ is the charge on site α of ion i and $\chi^0_{i\alpha}$ is the electronegativity of atom α. $J^0_{\alpha\alpha}$ and $J_{\alpha\beta}$ control the intramolecular contributions to the charge distribution whilst the fourth term controls the inter-molecular atom-atom interactions. A single parameter, $\Delta\chi = \chi^0_{iC} - \chi^0_{iO}$, effectively controls the molecular charge distribution with the larger the value of $\Delta\chi$ the greater the charge separation. The effect of varying the relative electronegativities is discussed in ref.[1]. In the present work we employ the parameter set which gives the charge distribution most consistent with experimental observation. The parameters are $\chi^0_O = 0.3213au$, $\chi^0_C = 0.2536au$, $J^0_{OO} = 0.4913au$, $J^0_{CC} = 0.4323au$, $J_{CO} = 0.2263au$, and $J_{OO} = 0.2903au$. In addition, the charge separation can be quantified by $\Delta q = q_C - q_O$, where q_C and q_O are the charges on the C and O atoms respectively.

Simulations are performed on systems containing 1296 atoms at fixed volume and temperature with the cell volumes obtained from the experimental densities[2–4] and temperatures maintained using Nosé-Hoover thermostats[5,6].

III. PARTIAL STRUCTURE FACTOR, $S_{CC}(k)$.

Figure S2 shows the carbon-carbon partial structure factor, $S_{CC}(k)$, which is used to determine the carbonate diffusion coefficient from the experimental scattering results. The figure shows a range of FCM results in which the charge distribution (characterised by Δq) is varied. The thick black line shows $S_{CC}(k)$ for the present work.

[1] M. Wilson, M.C.C. Ribeiro, M.C. Wilding, C. Benmore, J.K.R. Weber, O. Alderman, A. Tamalonis, and J.B. Parise, J. Phys. Chem. A **122** 1071 (2018)
[2] Janz,G.J. and Krebs,U. and Siegenthaler,H.F. and Tomkins,R.P.T., *J. Phys. Chem. Ref. Data*, **1**, 581 (1972).
[3] Janz,G.J. and Dampier,F.W. and Lakshminarayanan,G.R. and Lorenz,P.K. and Tomkins,R.P.T., *National Bureau of Standards Reference Data Series*, **15**, 1 (1966).
[4] Janz,G.J., *J. Phys. Chem. Ref. Data*, **17**, 1 (1988).
[5] S. Nosé, *J. Chem. Phys.*, **81**, 511 (1984).
[6] W. G. Hoover, *Phys. Rev. A*, **31**, 1695 (1985)

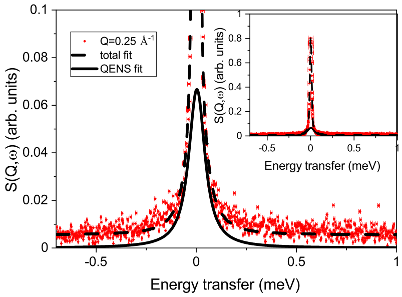
Q=0.25 Å^{-1}
total fit
QENS fit
S(Q,ω) (arb. units)
Energy transfer (meV)
S(Q,ω) (arb. units)
Energy transfer (meV)

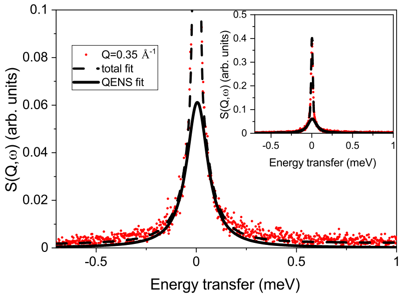
Q=0.35 Å^{-1}
total fit
QENS fit
S(Q,ω) (arb. units)
Energy transfer (meV)
S(Q,ω) (arb. units)
Energy transfer (meV)

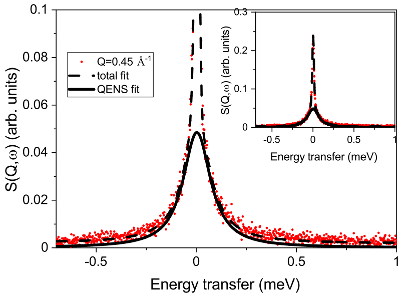
Q=0.45 Å^{-1}
total fit
QENS fit
S(Q,ω) (arb. units)
Energy transfer (meV)
S(Q,ω) (arb. units)
Energy transfer (meV)

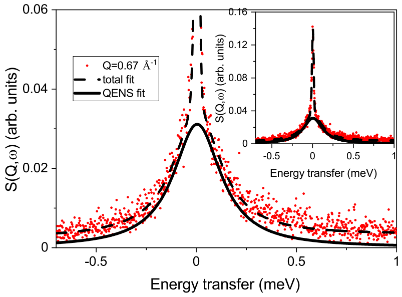
Q=0.67 Å^{-1}
total fit
QENS fit
S(Q,ω) (arb. units)
Energy transfer (meV)
S(Q,ω) (arb. units)
Energy transfer (meV)

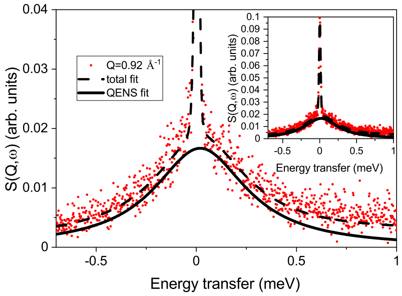
Q=0.92 Å^{-1}
total fit
QENS fit
S(Q,ω) (arb. units)
Energy transfer (meV)
S(Q,ω) (arb. units)
Energy transfer (meV)

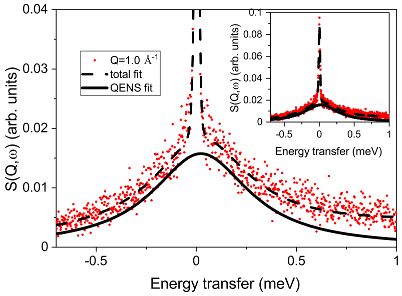
Q=1.0 Å^{-1}
total fit
QENS fit
S(Q,ω) (arb. units)
Energy transfer (meV)
S(Q,ω) (arb. units)
Energy transfer (meV)

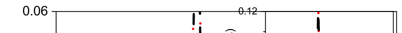

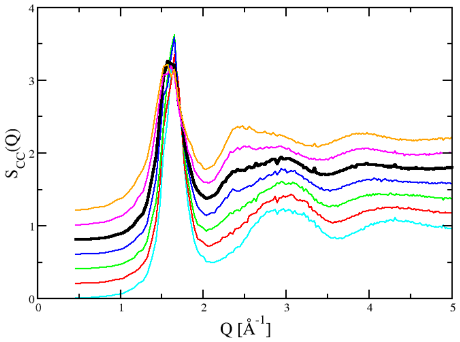

FIG. S2. Key: (from bottom to top) $\Delta q = -0.68, 0.20, 0.80, 1.28, 1.76, 2.24$, and $2.28e$.
